# Biology, Ecology, and Management of *Erthesina fullo* (Hemiptera: Pentatomidae): A Review

**DOI:** 10.3390/insects11060346

**Published:** 2020-06-03

**Authors:** Qianqian Mi, Jinping Zhang, Elaine Gould, Juhong Chen, Zhitan Sun, Feng Zhang

**Affiliations:** 1 MARA-CABI Joint Laboratory for Bio-safety, Institute of Plant Protection, Chinese Academy of Agricultural Sciences, Beijing 100193, China; q.mi@cabi.org (Q.M.); j.zhang@cabi.org (J.Z.); 15181674153@163.com (J.C.); 2 Zespri International Ltd., 400 Maunganui Road, Mt Maunganui 3149, New Zealand; Elaine.Gould@zespri.com (E.G.); Zhitan.Sun@zespri.com (Z.S.); 3 College of Agriculture and Ecological Engineering, Hexi University, Zhangye, Gansu 734000, China

**Keywords:** yellow spotted stink bug, host plant, crop damage, biological control, IPM, biosecurity risk

## Abstract

The yellow spotted stink bug (YSSB), *Erthesina fullo* Thunberg, is one of the most widely distributed phytophagous insect pests in Asia. YSSB is highly polyphagous and in China it feeds on over 57 host plants in 29 families, including some economically important fruit crops such as kiwifruit, pear, peach, apple, and pomegranate. With a primarily *r*-selected life history strategy, reproductive diapause, aggregation behavior, wide host range, high dispersal capacity, and close association with human-modified ecosystems, YSSB is a potentially invasive species that poses significant biosecurity threats to other countries outside its native range. This review summarizes basic and applied knowledge on the biology, ecology, and management of YSSB in China, with specific emphasis on its life history, host range, damage and impacts on economically important horticulture crops, and integrated pest management (IPM) approaches. The insights from the Chinese literature on this pest will help the countries outside its native range to conduct appropriate biosecurity risk assessments, develop a sound surveillance program, and develop an emergency response plan before its invasion of new geographic areas.

## 1. Introduction

The yellow spotted stink bug (YSSB), *Erthesina fullo* Thunberg (Hemiptera: Pentatomidae), is one of the most widely distributed phytophagous pests in Asia, including Bangladesh, China, India, Indonesia, Japan, Myanmar, Sri Lanka, and Vietnam [1,2,3,4,5]. It feeds on a number of economically important fruit, such as apples, cherries, pears, and kiwifruit [6,7,8,9,10]. Known as a hitchhiker pest, YSSB has been carried into other areas outside its current distribution in a variety of ways, including on passengers/luggage, containers, general cargo, used machinery, and vehicles [11]. In November 2014, a single female YSSB was discovered and destroyed in Temuka, New Zealand [12]. Although no further interceptions have since been reported there, YSSB is still considered as an important biosecurity risk to New Zealand [13].

In order to carry out proper risk analysis and develop corresponding pre-border, at-border, and post-border biosecurity activities to prevent the potential invasion of YSSB, more in-depth understanding of the biology and ecology of YSSB in its native range would provide useful local knowledge for researchers, quarantine officers, and industry biosecurity specialists in concerned countries. However, little is known about the biology and ecology of YSSB outside of Asia, although there are quite a few Chinese studies on this pest [13]. To assemble this information, extensive literature searches on YSSB were conducted in three Chinese databases: the Chinese National Knowledge Infrastructure, the Wanfang Data Knowledge Service Platform, and Weipu using Chinese characters. We also searched two international databases, Ovid and Web of Science, using English search terms (Table S1). After eliminating duplicate and irrelevant records, a total of 87 publications were identified, 76% of which were Chinese (Figure S1). We then summarized this information on YSSB with a particular focus on China, including its life history, host range, nature of damage, impacts on economically important horticulture crops, and integrated pest management approaches. The aim of this review is to provide a thorough assessment of YSSB biology, ecology and management, which should be useful for biosecurity risk assessment and the development of a surveillance program and emergency response plan before its introduction into new geographic areas.

## 2. Description of Life Stages

**Figure 1:**
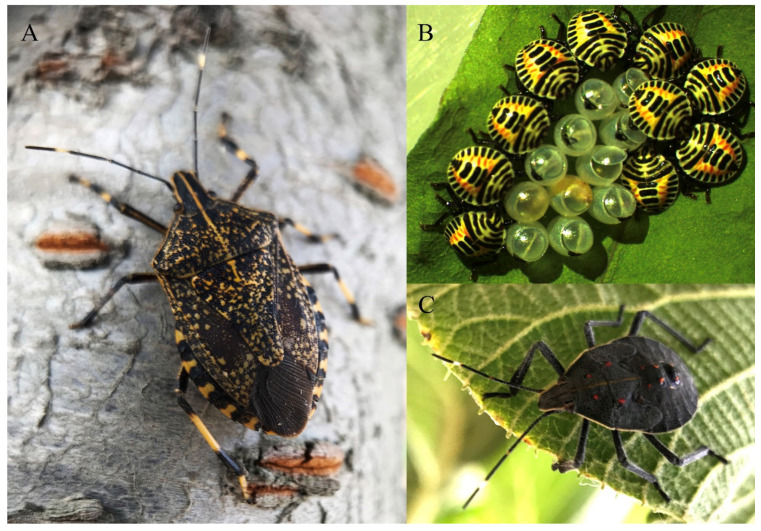
Life stages of *Erthesina fullo*. (**A**). adult; (**B**). eggs and first instars; (**C**). late instar nymph.

The adult of YSSB (Figure 1A) is slightly brownish black, and the body sizes (male: length 18–22 mm, width 8–10.5 mm; female: length 19–23 mm, width 9–11 mm) are larger than the brown marmorated stink bug (BMSB), *Halyomorpha halys* (Stål) [1,14]. The adult head is relatively big and tapering towards the front. There are some yellowish white dots between the red simple and black compound eyes. Antennae are black and filamentous with five segments, while the basal portion of the fifth segment is pale yellowish. A whitish yellow line runs back from the apex of the head, across the middle of the praescutum, and ends at the base of the scutellum. Both praescutum and scutellum are brownish black and covered by many small yellow spots. The anterior areas of the anterolateral margins of the pronotum are slightly serrated, and the forewing has small yellowish white spots. The rostrum reaches the third abdominal segment [1]. Females lay egg masses consisting of about 12 eggs (length approximately 2.9 mm, diameter 1.7 mm; Figure 1B), arranged more or less regularly [15]. Eggs are light greenish at the time of oviposition and become light brown and then faint yellow before hatching. The operculum is hemispherical, permitting the escape of the young nymph. The egg burster is chitinized, blackish, and triangle shaped. YSSB have five nymphal instars. The first instar (Figure 1B) is elliptical with red, white, and black stripes. There are three thick black stripes on the back of the abdomen. Later instars (Figure 1C) are reddish brown or dark brown with a yellow or yellowish-red vertical line from the front of the head to the scutellum. Antennae of the late instars are black with four segments. Four pale red spots are present in a horizontal row in the middle of the pronotum. There are three large dark spots on the back of the abdomen, and each spot has two pale red scent gland orifices [14].

## 3. Life History

YSSB is widely distributed across China, from north to south and from east to west, including Inner Mongolia, Liaoning, Beijing, Tianjin, Hebei, Henan, Shaanxi, Shanxi, Shandong, Anhui, Jiangsu, Shanghai, Zhejiang, Jiangxi, Hubei, Hunan, Sichuan, Guizhou, Yunnan, Guangdong, Guangxi, Hainan, and Taiwan [1,5]. YSSB is mostly univoltine (one generation per year) in northern China such as in Hebei, Henan, Shandong, and Shanxi provinces [10,15,16,17]. It has one to two generations per year on kiwifruit in Shaanxi province [8,9,15] and two generations per year in eastern China areas such as in Anhui and Jiangxi provinces [18,19]. YSSB is multivoltine in southern China, with three generations per year in Yunnan province [20]. 

Nonreproductive adults overwinter in both artificial and natural shelters such as dry crevices of buildings, tree holes, underneath fallen leaves or the bark of trees [21]. Univoltine populations gradually emerge from overwintering sites in mid-April, and begin laying eggs from late April to late June on kiwifruit in Shaanxi province (Table 1). First generation nymphs start hatching in mid-May and begin feeding on host plants through June and July. First generation adults occur in mid-August, with populations peaks in September, aggregating in kiwifruit orchards to feed on maturing fruits [15]. For bivoltine populations, the timing of YSSB emergence from overwintering sites in the spring is earlier than univoltine populations (Table 1). Peak populations of first-generation adults occur between late June and mid-August on kiwifruit, where they feed on young shoots, leaves, and fruit, while peak populations of second-generation adults occur from August through to October [8,9]. Thereafter, both univoltine and bivoltine YSSB adults begin moving to overwintering sites in mid-October and continue doing so into November.

Both reproductive and non-reproductive development of YSSB are closely related to photoperiod, temperature, and/or diet. YSSB adults leave overwintering sites in spring when temperatures exceed 13 °C [9]. Mating occurs throughout the day, especially between 12:00 and 14:00. The time needed for mating ranges from a few minutes up to three hours [15,20]. Multiple copulations are commonly observed for both males and females [18]. After mating, YSSB females start to oviposit in 1–2 days [20] or 3–5 days [18], the difference might be attributed to different geographic populations, ambient temperatures, or host plants. Eggs are typically laid on the undersides of leaves [15,18,20] or the base of kiwifruit [8]. The life-time fecundity of mated YSSB females was observed to be 126–173 eggs per female when maintained on caged branches of pomegranate, *Punica granatum* L. [20], but only 36–60 eggs per female on Chinese jujube, *Ziziphus jujuba* Mill. [18]. In a field cage study in mixed fruit crops, Zhang et al. [17] reported an average of 35 eggs per female (range of 12–68 eggs per female). Such a substantial variation in the lifetime fecundity of YSSB females might be attributed to both biotic and abiotic conditions during the testing period, including ambient temperature, food quality, insect physiological status, and sampling size.

YSSB eggs can complete their development from 15–30 °C and 75–95% relative humidity (RH), with a hatch rate exceeding 97.5%. However, the egg hatching rate was lower when the ambient temperature was less than 15 °C and the relative humidity was lower than 60% [20]. The mean developmental time of YSSB egg and nymphal stages at 25–30 °C and 80–95% RH was 5.5 d (4–7 d) and 27 d (21–33 d), respectively [20]. In a field cage study in Hebei province of northern China, the developmental time of YSSB eggs decreased with increased temperatures in the range of 18.15–26.12 °C on average and was 5–12 d between the end of May and mid-August [17]. The mean developmental time of 1st, 2nd, 3rd, 4th and 5th instars was 5.7 d (4–8 d), 14.9 d (12–21 d), 13 d (12–15 d), 13.2 d (12–18 d) and 18.5 d (16–22 d), respectively [17]. 

## 4. Dispersal Capacity and Behavior

After hatching, first instar YSSB nymphs remain aggregated on the egg mass for 5–10 h [20]. First and second instar nymphs exhibit aggregation behavior, but third instar nymphs disperse as individuals to seek food [20], however, there were also reports that second instars can disperse to seek food [8,15,18]. YSSB adults respond to light and often rest on the top kiwifruit vines and young fruit in the sunlight during the day [8,15], and then move to the undersides of leaves or other warmer places to rest during the night [18,20]. 

YSSB adults move from host plants to overwintering sites in the fall and then disperse again from overwintering sites the following spring. Aggregations of YSSB adults are found in overwintering sites, with dozens of individuals being together [15]. Although the dispersal behaviors of YSSB adults after exiting from overwintering sites are not well known, the movement of adults may be triggered by nutritional needs and the phenology of the host plant [18]. Field surveys have shown that YSSB disperses among different host plants [17]. In Shijiazhuang and Hengshui areas in Hebei province, higher numbers of stink bugs (including adults and nymphs of both YSSB and BMSB) were found on *Paulownia* spp. from mid-May through mid-July and thereafter decreased sharply, while the number of stink bugs increased dramatically on pear, *Pyrus bretscheider* Rehder, starting in mid-July. Relatively higher numbers of stink bugs were also observed on peach, *Prunus persica* (L.) in mid-May and then decreased sharply from the end of May to mid-August. However, the relative abundance of stink bugs varied only slightly on apple (*Malus pumila* Mill.) and poplar (*Populus* spp.) over time [17]. A field-based mark-recapture study also showed that overwintering YSSB adults can fly 3 km in five days in early spring [17]. Therefore, the dispersal capacity and behavior of YSSB adults and nymphs would have a profound effect on the distribution and population dynamics of YSSB among crops and landscapes during the crop growing season.

## 5. Host Range

YSSB is a polyphagous, arboreal species. Collectively, over 57 host plants in 29 families have been reported in China, with most hosts in the Rosaceae (Table 2). Its host range includes some economically important crops such as kiwifruit, pear, apple, peach, pomegranate, cherry, and Chinese jujube. It also feeds on woody ornamentals and wild plants such as black locust, Chinese plum, pine, paulownia, poplar, willow, and tree of heaven. Although YSSB has a wide range of host plants, it is not always a major pest depending on the region, host plant, and local population levels (Table 2). The relative abundance of YSSB on different host plants [17] might indicate that host plants vary in suitability and acceptability for YSSB. However, the development and fitness of YSSB on different host plants has rarely been studied.

Liu & Liu [22] reported that kiwifruit is not the most preferred host plant based on the observation that the numbers of YSSB on kiwifruit were less than on other host plants from the end of June to the end of September and that fewer eggs were laid on kiwifruit compared to other plants. This conclusion might be biased, as YSSB can disperse between host plants and across the landscape [17] and YSSB prefers to inhabit arboreal hosts, such as tree of heaven, during the daytime [22]. Moreover, Zhang [9] reported that the numbers of YSSB nymphs and adults were much higher than the numbers of BMSB between June and October in a kiwifruit orchard in Zhouzhi county, Shaanxi province, and suggested that YSSB was a dominant stink bug pest on kiwifruit in the surveyed area.

## 6. Damage and Impact

YSSB nymphs and adults feed on leaves, flowers, shoots, and fruit of various host plants [15]. Similar to BMSB and other pentatomids, YSSB inserts its stylet into the plant tissue for feeding and secretes a thick saliva [17] to break down tissue cells and enable the consumption of the liquified contents [35]. YSSB feeding results in discoloration, appearance of yellowish-brown spots, withering and even defoliation of leaves or shoots [33]. Feeding can cause dry, corky tissues just below the surface of feeding sites on fruit, which can harden, depressing and distorting the surface of the fruit, thereby reducing the fruits’ value [17,33]. In the worst cases, feeding can also cause fruit to abort prematurely, leading to significant yield losses [16]. In addition to direct damage, YSSB feeding can cause infestations of pathogenic bacteria or fungi, resulting in fruit rot [10,20]. However, unlike BMSB [36], YSSB is not able to transmit the phytoplasma responsible for Paulownia witches’ broom disease [37]. The following is a summary of the damage symptoms and impact of YSSB feeding injury to some economically important crops in China.

### 6.1. Kiwifruit

Besides BMSB, YSSB is another important stink bug pest on kiwifruit [8,9,13,15]. Both adults and nymphs of YSSB feed on leaves, flowers, buds, vines, and fruit [15], causing leaf yellowing, flower-, bud-, and fruit-drop or fruit deformation, respectively [8,22]. The injured fruit first hardens but then becomes soft in 3–5 d and drops to the ground 5–7 d after feeding, at which time the feeding site expands to a large circular spot (ca. 1 cm diameter) on the fruit surface and the flesh underneath the spot turns from dark green into light green with a bitter taste [15]. As such, YSSB feeding affects both fruit quality and storage [15,22]. Wang & Kang [15] reported that YSSB caused 10–30% yield losses and economic losses of CNY 100–200 (at the money value in 1999) per mu (1 ha equal to 15 mu, USD 181–362 per ha) in kiwifruit orchards, Zhouzhi county, Shaanxi province. Liu & Liu [22] reported that fruit injury levels were not significantly different among early (Cuixiang), mid- (Qinmei), and late (Hayward) maturing cultivars in a mixed kiwifruit orchard (25 ha), ranging from 4.1% to 48.1%. The mean fruit injury rate for Cuixiang, Qinmei and Hayward was 28.3%, 23.2%, and 30.6%, respectively. The number of feeding spots per damaged fruit ranged from 34 to 89. Severe fruit injury occurred more frequently in the rows of vines close to village roads (planted with tree of heaven) than in the central part of the kiwifruit orchard [22]. This strong edge effect is likely due to the polyphagous nature of YSSB and its movement between host plants (i.e., between kiwifruit and tree of heaven) to meet its nutritional or other physical requirements.

Damage in a kiwifruit orchard in Shaanxi province caused by a univoltine population of YSSB from nymphs and adults mostly occurred in June-July and September, respectively [15]. By contrast, damage caused by the bivoltine YSSB adults in Shaanxi province mostly occurred from the end of June to mid-August and from early August to mid-October for 1st and 2nd generations, respectively, with the fruit damage from 2nd generation YSSB being more serious [9]. 

### 6.2. Pear

Both YSSB and BMSB are regarded as key pests of pear in Hebei province [7,17] along with the hairy shieldbug, *Dolycoris baccarum* (L.), in Anhui province [6]. The damage rate of stink bugs was observed to be 5–10% in most pear orchards, 20–30% in more severely attacked pear orchards [6], and as high as 40–60% in the most heavily attacked orchards [17]. The collective literature did not separate the damage between stink bug species, as they mostly occur at same time during the pear growing season. Xu & Jiang [6] reported that YSSB adults caused the most damage to pear fruit between the end of June and mid-July.

### 6.3. Other Crops

Crop damage by YSSB has also been reported in other economically important crops in China. YSSB attacked pomegranate flowers and fruit, with injury rates commonly reaching 12–15% and 15–20%, respectively [20]. A fruit injury rate of 10–25% by YSSB was likewise reported on Chinese jujube [18]. YSSB, BMSB, and the pear stink bug, *Urochela luteovaria* Distant (Hemiptera: Urostylidae) were reported as the three major stink bug pests of apple, with the fruit injury rate caused by these stink bug pests reaching 60% [10]. As an introduced plant in China since the 1960–1970s, macadamia nut trees were seriously attacked by several stink bug pests including YSSB and BMSB, with nut damage exceeding 40% and even reaching 90% in poorly managed orchards in Guangxi province [29]. Feeding by stink bugs on macadamia nut trees can result in shrinking or rotting nuts with no or significantly reduced market value.

### 6.4. Nuisance Problem

As adults aggregate and overwinter in large numbers inside or under the roofs of human dwellings [15,17,18], YSSB has the potential to be an important nuisance pest like BMSB [38]. YSSB also emits distinctive odor when disturbed [1].

## 7. Integrated Pest Management Approaches

### 7.1. Sampling and Monitoring

Visual observation is the primary sampling method used to assess emergence patterns, relative population density and seasonal phenology of YSSB field populations [15,17,20,23,31]. Zhang [9] used sweep nets to measure the population dynamics of stink bug pests between the end of June and early October on kiwifruit, and found YSSB captures peaked on the 10th of July (with 3.4 stink bugs per 10 sweeps of a net) and the 7th of September (with 4.6 stink bugs per 10 sweeps of a net). Zhang [9] also used four different colored sticky traps (25 cm × 30 cm per trap, 20 replicates per color) on kiwifruit to compare their efficiencies. The maximum mean YSSB catch was recorded with the bluish-green sticky traps (with 0.6 stink bugs per trap per 5 days) followed by blue and yellow traps, with the fewest caught in green traps (with 0.3 stink bugs per trap per 5 days). Due to the lower efficiency and costs of colored sticky traps, the sweep net was recommended to be used for sampling and monitoring of YSSB in the field [9]. Beat sampling would also work because of the dropping behavior of disturbed YSSB, although this has not yet been reported to be used. Based on our own experiences with BMSB, beat sampling is difficult to implement on kiwifruit as the vines are fixed and it is hard to beat/shake vines to make stink bugs drop from them.

### 7.2. Cultural Control

Sanitation is one of the most commonly recommended cultural control methods used to control YSSB in China. During the winter, sanitary measures such as clearing ground-covering vegetation within or near the orchard, fallen leaves and vines, and removal of dry bark has been suggested [6,15,20,22]. Sanitation measures should also be applied to the fruit storage facility, human dwellings or other artificial structures within or around the kiwifruit orchards to remove and kill the overwintering YSSB adults [15]. Other sanitation measure includes destruction of alternate hosts within or near the orchard [8]. A common pest control practice used on ornamental trees, painting trunks with lime water, lime-sulfur solution, or Bordeaux mixture, could be used in kiwifruit or pomegranate to destroy the overwintering sites and suppress YSSB overwintering populations [20,22].

### 7.3. Physical Control

According to YSSB’s aggregation behavior in overwintering sites in the fall, trapping with a bunch of straw attached to the base of the trunk or branch/vine can be deployed to aggregate overwintering adults and then kill them by burning or otherwise disposing of the straw [8,16,22]. Song & Wang [16] also recommended this trapping method for a Chinese jujube orchard with 2–3 trees per mu and 1–2 bunches of straws per tree depending on the population density of YSSB during the season.

Bagging of individual fruit has also been recommended as an effective control method to avoid feeding injury by YSSB [22,31]. Liu & Liu [22] stated that feeding injury rate on kiwifruit fruit in bags was significantly reduced compared to orchards without bagging. Zhang et al. [31] also confirmed a significant control effect of bagging on apple, peach, pear, and apricot from damage by stink bugs (including YSSB and BMSB), with damage being reduced from 37.5%–62.0% in non-bagged fruit to 5.9%–8.6% for bagged fruit. However, both YSSB and BMSB have long stylets, and can still pierce fruit through the bags to feed on apples, pears, or peaches inside the bag [10,17]. Therefore, it was highly recommended to use a relatively larger bag to cover kiwifruit and apples [22,31]. Although fruit bagging is widely practiced in China, it is very labor intensive and the implementation costs are higher than other practices such as insecticide spraying. For example, the fruit bagging practice costs about CNY 19,500 per ha (USD 2,700 at the value in 2020) for kiwifruit growers.

Other physical control methods include mechanical removal of eggs and nymphs from crops [6,8,18,22,23], removal of overwintering adults when they disperse into or from overwintering sites such as human dwellings, agricultural machinery rooms or other artificial shelters [8,16,17,31], and removal of YSSB by shaking or beating vines in early spring, during early morning or evening because of their dropping behavior [8,22,33]. However, these physical removal methods are time consuming and costly, which might not be efficient for growers with large areas of fruit plantations. 

Using light traps has also been mentioned as a control method [18,39], yet some studies have found YSSB nymphs and adults to have very weak orientation tendency towards light [33]. Due to these contradictory reports, the usefulness of light trapping is debatable. 

### 7.4. Host Plant Resistance

The crop itself is the starting point for any IPM system, and thus host plant resistance can often play a fundamental role in IPM [33]. Liu & Liu [22] found that YSSB did not have any feeding preference and caused similar feeding injury among three kiwifruit cultivars, namely Cuixiang, Qinmei, and Hayward. The feeding preferences of YSSB on peaches and Chinese jujubes have likewise been reported [16,32,40]. In a germplasm nursey in Henan province, varieties of nectarines (*Prunus persica var. nectarina*), flat peaches (*Amygdalus persica* L. ‘Compressa’) and yellow-fleshed peaches (*Amygdalus persica* L.) were all highly susceptible to YSSB, while varieties of white peaches and ornamental flowering peaches were somewhat susceptible, and varieties of dwarf peaches (*Amygdalus persica* L. *var. densa* Makino) showed resistance with no fruit injury by YSSB [32,40]. Pest resistance levels also differed among peach varieties within the same group, and it seemed the difference was depending on whether or not the fruit skin had fuzz. For example, those varieties without fuzz in nectarine groups were heavily attacked by YSSB, whereas those varieties with fuzz in ornamental flowering peach groups were much less attacked [32,40]. In addition, a field cage study on different Chinese jujube varieties showed that YSSB preferred to feed more on “Hui-zao” than on “Ji-xin-zao” or “Jiu-yue-qin,” and the mortality of YSSB nymphs or adults was much higher in the latter two resistant varieties [16]. 

### 7.5. Biological Control

Biological control can be a cost-effective, sustainable, and environmentally safe approach for the long-term management of arthropod pests. There are only a few reports on natural enemies of YSSB in China, including parasitoids, predators and entomopathogens (Table 3). Hymenopteran egg parasitoids are the primary natural enemy of YSSB, but parasitism rates are highly variable among different habitats and studies (Table 3) [17,19,22,23]. Liu & Liu [22] reported that the parasitism rate of YSSB in pear and apple orchards reached 70–80%, and thus predicted that biological control with mass release of egg parasitoids would effectively control YSSB in kiwifruit orchards even though there was no field release data to support such an optimistic assumption. A field study in Hebei province showed that *Trissolcus flavipes* Thomson (Hymenoptera: Scelionidae) was a dominant parasitoid of YSSB eggs in pear and peach orchards, and the mean and maximum parasitism rate between May and August was 30.6% and 43.5%, respectively [17]. Wang & Qiu [19] reported that *Anastatus fulloi* Sheng et Wang (Hymenoptera: Eupelmidae) and *Telenomus* sp. (Hymenoptera: Scelionidae) were the two dominant parasitoids attacking 1st generation YSSB eggs on uncultivated host plants, with a parasitism rate of 28.9% and 27.1%, respectively. As for the 2nd generation YSSB eggs, *Ootetrastichus* sp. (Hymenoptera: Eulophidae) was the dominant parasitoid with a mean parasitism rate of 42.5%, followed by *A. fulloi* (21.2%), *Telenomus* sp. (10.8%) and *Mesopolobus tabatae* (Ishii) (Hymenoptera: Pteromalidae) (3.8%). In contrast to the above studies, Su [23] reported only 2% parasitism of YSSB eggs in the field from a 3-year study in Xi county, Henan province. Since mass rearing techniques of *Anastatus* species have been well established in China [41], there is higher potential to use *A. fulloi* with an augmentative biological control approach to attack YSSB eggs, and thereby reduce YSSB nymphal populations in the field.

The literature on predators and entomopathogens of YSSB is very limited. Su [23] made a very general statement that birds such as the black drongo, great tit, and magpie could play an important role in reducing YSSB nymphs in fruit orchards. Interestingly, Lu et al. [18] found that *Beauveria bassiana* (Bals.) Vuill. infested YSSB adults and the infestation rate was 20–30% in Chinese jujube orchard during the rainy season in June in Anhui province.

The defense behavior of stink bugs is a consideration in biological control. When disturbed, YSSB release defensive chemicals that may deter natural enemies [33]. Laboratory behavioral bioassays showed that the odor of YSSB male metathoracic scent gland elicited an alarm response, making the conspecific male individuals alert and disperse [28]. Kou et al. [28] also identified nine compounds from the glandular secretion of both YSSB males and females, including (*E*)-2-hexenal, (*E*)-4-keto-2-hexenal, (*E*)-2-hexenyl acetate, *n*-undecane, *n*-dodecane, (*E*)-2-decenal, *n*-tridecane, (*E*)-2-decenyl acetate, and *n*-pentadecane. Although the biological functions of these compounds need to be further clarified, understanding the behavioral and chemical ecology of YSSB and its interactions with parasitoids or predators would help develop novel methods to enhance our biological control attempts. 

### 7.6. Chemical Control

Chemical control is currently the most widely used control method for YSSB in China [33]. A list of insecticides recommended against YSSB is summarized in Table 4, including organophosphates and pyrethroids. The efficacy of some insecticides against YSSB was tested either in the laboratory or in the field. The mortality rate of YSSB nymphs reached 100% in 24 or 48 h after their exposure to some of the insecticides in the laboratory, e.g., malathion, omethoate, deltamethrin, fenpropathrin and lambda-cyhalothrin [23]. Field cage tests on Chinese jujube showed that isocarbophos and omethoate provided nearly 100% and over 95% control of YSSB in 48 h after spraying, respectively [16]. Zhang et al. [31] reported that insect growth regulators such as chlorbenzuron and triflumuron applied in the field provided over 90% and 80% control of YSSB nymphs and adults, respectively. In a 100-ha pomegranate orchard, the damage rate to flower-buds and fruit from YSSB was reduced from 12–15% and 5–7% to 5–7% and 3–5%, respectively, after spraying fenvalerate, lambda-cyhalothrin, omethoate, dichlorvos, or methidathion [20]. Besides the use of pesticide sprays to control YSSB, Feng [8] suggested fumigation with a combination of sawdust, ammonium nitrate, diesel, and dichlorvos in the center of a kiwifruit orchard in late evening or early morning of a cloudy day. Fumigation was suggested to control YSSB overwintering adults in the barns and other non-occupied buildings surrounding fruit orchards [31]. Moreover, ‘Quchunwang’, a local chemical product with repellent effect towards YSSB, could be applied to vines in early June at the ratio of 40–60 pieces per mu, which would last for 46–87 day with repellent effect of approximately 70–96.4% [43].

Timing of insecticide applications is an important factor affecting chemical control efficacy against YSSB. Wang & Kang [15] suggested applying insecticides at different crucial time periods in kiwifruit production, timed to control overwintering YSSB adults, eggs, and 1–2 instar nymphs. Others have suggested targeting insecticide treatments against YSSB nymphal populations rather than other life stages [8,16,23]. However, the recommended chemical insecticides, except for insect growth regulators, are highly toxic to bees (Table 4) and probably other natural enemies as well. Therefore, to protect natural enemies in the field, Zhang et al. [31] suggested applying insect growth regulators rather than broad-spectrum insecticides during the peak time of YSSB occurrence.

### 7.7. Area-wide Control Approach

As mentioned above, YSSB adults can disperse between host plants and across the landscape. Through field surveys, Song & Wang [16] reported that the density of YSSB is much higher in mixed fruit orchards of Chinese jujube and persimmon/pear/apricot than pure Chinse jujube orchards. Moreover, higher densities of YSSB were observed on plants in edge rows than those in the central area of the orchards [16]. Such a strong edge effect also occurs in kiwifruit orchards, and the association of YSSB with adjacent forested areas and other highly preferred host plants may drive this border effect [22]. Based on the phenological information of YSSB, Zhang et al. [17] suggested the use of area-wide measures to improve control effects, applying chemical control mainly on paulownia and peach crops in June–July and then on pear crops in July–August. Therefore, an area-wide control approach was recommended to manage YSSB not only on target crops (e.g., Chinese jujube, pear, kiwifruit) but also on host plants in surrounding areas [16,22,31].

## 8. Conclusions

YSSB is an important stink bug pest in China, causing economic damage to many horticultural crops such as kiwifruit, pear, peach, apple, pomegranate, and Chinese jujube, although actual economic losses in those crops are not always well documented. Interestingly, there are over 14 Chinese publications discussing both YSSB and BMSB, indicating their similarities or overlaps regarding the ecological niche, field occurrence, host plants, and/or control measures. Although YSSB’s reproductive output is lower than BMSB, the two sympatric species still share many other biological characteristics, e.g., polyphagy, a primarily *r*-selected life history, reproductive diapause, high dispersal capacity, aggregation behavior, and association with human-modified ecosystems, which make the latter a successful global invader [38]. BMSB has already established in the United States, Canada, many European countries and Chile, and become a key agricultural pest causing significant damage and losses to tree fruit, small fruit, nuts, and vegetables [38]. YSSB could also become an ecological and economic danger to other parts of the world outside its’ area of origin if proper conditions and circumstances arise [45]. As far as New Zealand is concerned [13,46], and based on the mapping study with BMSB [47], there is no doubt that YSSB could establish in the country if it arrives and would potentially have significant economic impacts on a range of important horticultural crops, such as stone fruit, apples, and kiwifruit.

Current control programs for YSSB in cultivated crops in China rely primarily on chemical insecticides, which are mostly conventional broad-spectrum products and not always compatible with IPM programs. The control effects of insecticide treatments may be reduced because abundant wild host plants serve as reservoirs for this highly polyphagous and mobile pest, allowing it to reinvade treated crops. Moreover, extensive and indiscriminate usage of these broad-spectrum insecticides would result in environmental degradation, adverse effects on human health and other organisms, residue problems in the agricultural produce, eradication or reduction of pollinators and natural enemies, and the development of resurgence and resistance to pesticides in insect pests. It is therefore very important to explore more specific and environmentally friendly control methods and apply a biologically-based, area-wide IPM strategy against YSSB as well as other stink bugs such as BMSB. Biological control agents such as *A. fulloi* and *B. bassiana* should be further investigated to explore augmentative biological control as a control option, which could play an important role in an IPM strategy for sustainable management of YSSB.

## Figures and Tables

**Table 1:** Seasonal phenology of univoltine and bivoltine populations of *Erthesina fullo* on jujube and kiwifruit in northern China.

Host Plant/Location	Jan	Feb	Mar	Apr			May			Jun			Jul			Aug			Sep			Oct			Nov	Dec
				E	M	L	E	M	L	E	M	L	E	M	L	E	M	L	E	M	L	E	M	L		
Jujube/Henan ^1^	(+)	(+)	(+)	(+)	+	+	+	+	+	+	+	+	+	+	+	+	+	+								
										●	●	●	●	●	●	●	●	●	●							
										−	−	−	−	−	−	−	−	−	−	−	−	−				
											+	+	+	+	+	+	+	+	+	+	+	+	+	(+)	(+)	(+)
Kiwifruit/Shaanxi ^2^	(+)	(+)	(+)	(+)	+	+	+	+	+	+	+	+	+	+	+	+	+	+								
						●	●	●	●	●	●	●														
								−	−	−	−	−	−	−	−	−										
																	+	+	+	+	+	+	(+)	(+)	(+)	(+)
Kiwifruit/Xi’an, Shaanxi ^3^	(+)	(+)	+	+	+	+	+	+	+	+	+	+														
							●	●	●	●	●	●														
							−	−	−	−	−	−	−	−												
											+	+	+	+	+	+	+									
											●	●	●	●	●											
													−	−	−	−	−	−								
																+	+	+	+	+	+	+	(+)	(+)	(+)	(+)

^1^ Univoltine population reported by Song & Wang [16]. ^2^ Univoltine population reported by Wang & Kang [15]. ^3^ Bivoltine population reported by Feng [8], and Zhang [9]. E: The early 10 days of a month. M: The middle 10 days of a month. L: The last 10 days of a month. (+) Overwintering adult, + Adult, − Nymph, ● Egg.

**Table 2:** Host plants and pest status of *Erthesina fullo* reported in China.

Family	Common Name	Scientific Name ^1^	Pest Status ^2^	Reference
Actinidiaceae	Kiwifruit	*Actinidia chinensis* Planch	A major pest of kiwifruit in Shaanxi province	[8,9,13,15,22]
Amaranthaceae	Beet	*Beta vulgaris* L.	-	[1]
Brassicaceae	Rape	-	-	[1]
Cupressaceae	Dawn redwood	*Metasequoia glyptostroboides* Hu & W. C. Cheng	A pest of dawn redwood in Henan province	[23]
Ebenaceae	Persimmon	*Diospyros* spp.	A pest of persimmon in Hebei and Henan provinces	[7,23]
Euphorbiaceae	Castor oil plant	*Ricinus communis* L.	-	[1]
	Chinese tallow tree	*Sapium sebiferum* (L.) Roxb.	-	[1]
	Chinese wood-oil-tree	*Vernicia fordii* (Hemsl.) Airy Shaw	-	[1]
Fabaceae	Black locust	*Robinia pseudoacacia* L.	A pest of black locust in Hebei and Henan provinces	[7,23]
	Chinese scholar tree	*Sophora japonica* L.	A major pest of Chinese scholar tree in Hebei province	[17]
	Silk tree	*Albizia julibrissin* Durazz.	A pest of silk tree in Hebei and Henan provinces	[7,23]
Fagaceae	Chinese chestnut	*Castanea mollissima* Bl.	-	[5]
Juglandaceae	Chinese wingnut	*Pterocarya stenoptera* C. DC.	A pest of Chinese wingnut in Shaanxi province	[22]
Leguminosae	White clover	*Trifolium repens* L.	-	[24]
Moraceae	Fig	*Ficus carica* L.	-	[10]
	Mulberry	-	-	[1]
	Paper mulberry	*Broussonetia papyrifera* (L.)	-	[25]
Myrtaceae	Clove	*Syzygium aromaticum* (L.) Merr. & L. M. Perry	-	[14]
	Guava	*Psidium guajava* L.	-	[20]
Oleaceae	Chinese ash	*Fraxinus chinensis* Roxb.	A pest of Chinese ash in Hebei and Jilin provinces	[7,26]
	Chinese privet	*Ligustrum lucidum* Ait.	-	[25]
	Wild forsythia	*Forsythia* spp.	-	[27]
Pinaceae	Pine	-	A major pest of pine trees in Taiwan	[28]
Platanaceae	Planetree	*Platanus* spp.	A pest of planetree in Hebei province	[7]
Poaceae	Bamboo	-	A pest of bamboo in Henan province	[23]
	Sugarcane	*Saccharum officinarum* L.	-	[1]
Punicaceae	Pomegranate	*Punica granatum* L.	One of the main pests of pomegranate in Yunnan and Henan provinces	[1,5,20,23]
Proteaceae	Macadamia nut	*Macadamia ternifolia* F. Muell.	A major pest of macadamia nut in Guangxi province	[29]
Rhamnaceae	Chinese jujube	*Ziziphus jujuba* Mill.	A pest of Chinese jujube in Anhui and Henan provinces	[18,23]
Rosaceae	Apple	*Malus pumila* Mill.	A common pest feed on apple in Hebei, Shandong and Shaanxi provinces	[10,15,17,30,31]
	Apricot	*Prunus armeniaca* L.	A common pest feed on apricot in Hebei, Henan and Shaanxi provinces	[15,23,30,31]
	Cherry	*Prunus* spp.	A pest of cherry in Henan province	[23]
	Chinese flowering crabapple	*Malus* spp.	A pest of Chinese flowering crabapple in Hebei province	[7]
	Chinese pearleaf crabapple	*Malus asiatica* Nakai	-	[1,5]
	Chinese hawthorn	*Crataegus pinnatifida* Bunge	A pest of Chinese hawthorn in Henan province	[23]
	Chinese plum	*Armeniaca mume* Sieb	-	[1,5]
	Japanese cherry	*Cerasus* spp.	A pest of Japanese cherry in Hebei province	[7]
	Loquat	*Eriobotrya japonica* (Thunb.) Lindl.	-	[20]
	Peach	*Prunus persica* (L.)	A major pest of peach in Hebei, Henan and Shaanxi provinces	[15,17,23,30,31,32] ^3^
	Pear	*Pyrus bretscheider* Rehder *Pyrus bretscheider* Rehder f. Ya-li Yü *Pyrus bretscheider* Rehder f. Hsüeh-hua-li Yü *Pyrus bretscheider* cv. Dangshansu pear	A major pest of pear in Hebei, Henan, Shaanxi and Anhui provinces	[6,15,17,23,30,31]
	Plum	*Prunus* spp.	A pest of plum in Henan province	[23]
	Quince	*Cydonia oblonga* Mill.	-	[1]
Rutaceae	Citron	*Citrus medica* L.	-	[33]
	Citrus	-	-	[1,5]
	Pomelo	*Citrus maxima* (Burm) Merr.	-	[20]
Salicaceae	Chinese white poplar	*Populus tomentosa* Carriere	A pest of Chinese white poplar in Shaanxi province	[22]
	Black poplar	*Populus nigra* L.	A pest of black poplar in Henan province	[34]
	Poplar	*Populus* spp.	A pest of poplar in Hebei and Shaanxi provinces	[15,17]
	Weeping willow	*Salix babylonica* L.	-	[1]
	Willow	*Salix* spp.	A pest of willow in Hebei, Henan and Shaanxi provinces	[15,17,23]
Sapindaceae	Longan	*Dimocarpus longan* Lour.	-	[1,5]
Sapotaceae	Nato tree	*-*	A major pest of nato tree in Taiwan	[28]
Scrophulariaceae	Paulownia	*Paulownia* spp.	A major pest of paulownia in Hebei and Henan provinces	[17,23]
Simaroubaceae	Tree of heaven	*Ailanthus altissima* (Mill.) Swingle	-	[1]
Solanaceae	Tobacco	-	-	[1]
Sterculiaceae	Chinese parasol tree	*Firmiana simplex* (L.) W. Wight	-	[1]
Ulmaceae	Elm	*Ulmus pumila* L	A major pest of elm in Hebei and Henan provinces	[17,23]
Vitaceae	Grape	*Vitis* spp.	A pest of grape in Henan province	[23]

^1^ Scientific name is not included when it was not clearly mentioned in the cited reference. ^2^ The pest status is only included when YSSB was clearly indicated as a pest of a host plant in the literature. ^3^ [32]: 201 of 208 peach varieties selected were attacked by YSSB in a germplasm nursery.

**Table 3:** List of potential natural enemies against *Erthesina fullo* identified in China.

Agent	Family	Host Stage Affected	% Parasitism ^1^	Reference
**Parasitoids**				
*Anastatus fulloi* Sheng et Wang	Eupelmidae	Egg	28.9%	[19]
*Mesopolobus tabatae* (Ishii)	Pteromalidae	Egg	3.8%	[19]
*Ootetrastichus* sp.	Eulophidae	Egg	42.5%	[19]
*Telenomus gifuensis* Ashmead	Scelionidae	Egg	2%	[23]
*Telenomus* sp.	Scelionidae	Egg	27.1%	[19]
*Trissolcus flavipes* Thomon (syn. *Trissolcus cultratus* Mayr)	Scelionidae	Egg	43.5%	[17]
*Trissolcus japonicus* (Ashmead) (syn. *Trissolcus halyomorphae* Yang)	Scelionidae	Egg	-	[42]
*Trissolcus* sp.	Scelionidae	Egg	2%	[23]
**Predators**				
Birds	-	Nymph and adult	-	[20,33]
Black drongo (*Dicrurus macrocercus* Vieillot)	Dicruridae	Nymph and adult	-	[23]
Great tit (*Parus major* L.)	Paridae	Nymph and adult	-	[23]
Magpie (*Pica pica* L.)	Corvidae	Nymph and adult	-	[23]
Chickens	Phasianidae	Nymph and adult	-	[20]
Mantis	-	Nymph	-	[33]
Spider	-	Nymph and adult	-	[33]
**Entomopathogens**				
*Beauveria bassiana* (Bals.) Vuill.	Cordycipitaceae	Adult	30%	[18]

^1^ % parasitism (either maximum or mean) in the field is included when it was clearly indicated in original paper.

**Table 4:** List of insecticides used against *Erthesina fullo* in China.

Insecticide Class	Active Ingredient	Host Stage Affected	Preharvest Interval (Days) ^1^	Toxicity to Bees ^2^	Reference
Carbamates	Metolcarb	Adult	25	Highly toxic	[15]
Insect growth regulator (Benzoylureas)	Chlorbenzuron	Nymph	7	Relatively non-toxic	[31]
	Triflumuron	Nymph	21	Relatively non-toxic	[31]
Organophosphates	Acephate ^3^	Egg	45	Highly toxic	[15]
	Chlorpyrifos ^3^	Nymph	28	Highly toxic	[31]
	Dichlorvos	Nymph, adult	7	Highly toxic	[15]
	Fenitrothion	Nymph	15	Highly toxic	[15]
	Isocarbophos ^3^	Egg, nymph, adult	28	Highly toxic	[15,16]
	Malathion	Nymph, adult	10	Highly toxic	[15,23]
	Methidathion	Adult	30	Highly toxic	[15]
	Omethoate ^3^	Nymph, adult	21	Highly toxic	[15,23]
Pyrethroids	Alpha-cypermethrin	Nymph	14	Highly toxic	[33]
	Beta-cypermethrin	Nymph	40	Highly toxic	[33]
	Bifenthrin	Nymph	10	Highly toxic	[17]
	Deltamethrin	Nymph	14	Highly toxic	[15,23]
	Fenpropathrin	Nymph, adult	14	Highly toxic	[15,17,23]
	Fenvalerate	Nymph, adult	20	Highly toxic	[15,16]
	Lambda-Cyhalothrin	Nymph	7	Highly toxic	[23,33]
Repellent	Quchunwang ^4^	Adult	Unknown	Unknown	[43]

^1^ Maximum preharvest day is used if it is different days between different host crops. ^2^ Toxicity to bees: highly toxic (LD_50_ < 2 μg a.i./bee); moderately toxic (LD_50_ 2–11 μg a.i./bee); relatively non-toxic (LD_50_ > 11 μg a.i./bee) [44]. ^3^ Chemicals currently in phase-out process and not permitted to apply on vegetables, oranges, and/or other fruit plants in China. ^4^ Product name only was reported without information on active ingredient.

## Supplementary Materials

The following are available online at https://www.mdpi.com/2075-4450/11/6/346/s1, Table S1: Literature searches for the yellow spotted stink bug, *Erthesina fullo*, in Chinese and English. Figure S1: Number of publications on *Erthesina fullo* published by decade in Chinese and English.
