# Supplementary material for: Biology, Ecology, and Management of Erthesina fullo (Hemiptera: Pentatomidae): A Review
Source: Insects. 2020 Jun 3;11(6):346. doi: 10.3390/insects11060346 (PMC7349651; doi:10.3390/insects11060346)
Supplement: Supplementary file 1 [file insects-11-00346-s001.pdf]

Supplementary materials

Table S1. Literature records searched for the yellow spotted stink bug, *Erthesina fullo,* in Chinese and English.

Platform	Databases (and extent of coverage)	Search terms in the 'subject' field	Search options	Search results (No. publications)
Chinese National Knowledge Infrastructure	China Academic Journal Network Publishing Database (1951- >), PhD Dissertations Database (1984- >), Master Dissertations Database (1989- >), Domestic Conference Database (1962- >)	‘麻皮蝽’或‘黄斑蝽’或‘黄霜蝽’ 或‘麻蝽象’ 或‘麻纹蝽’	'Subject'	56
Wanfang Data Knowledge Service Platform	China Online Journals (1998- >), China Dissertations Database (1980- >), China Conference Proceedings Database (1982- >)	‘麻皮蝽’ 或‘黄斑蝽’ 或‘黄霜蝽’ 或‘麻蝽象’ 或‘麻纹蝽’	'Subject'	69
Weipu	China Science and Technology Journal Database (1992- >)	‘麻皮蝽’ 或‘黄斑蝽’ 或‘黄霜蝽’ 或‘麻蝽象’ 或‘麻纹蝽’	'Topic' 'Keyword'	26
Ovid	AGRICOLA (1970-2020), BIOSIS Previews (1987-2011), CAB Abstracts (1973- >), CAB Abstracts Archive (1910-1972), Econlit (1886- >), ERIC (1965-2019), Food Science and Technology Abstracts (1969- >), Ovid MEDLINE(R) and Epub Ahead of Print, In Process & Other Non-Indexed Citations and Daily (1946- >), Ovid Mediline(R) (2015- >), Zoological Record (2001-2007)	'Yellow marmorated stink bug' OR '*Erthesina fullo*' OR 'Yellow spotted stink bug' OR 'YSSB' OR 'YMSB'	'Keyword'	46
Web of Science	WoS Core Collection (1900- >), CAB Abstracts (1973-- >), Chinese Science Citation Database (1989- >), Derwent Innovation Index (1963- >), KCI-Korean Journal Database (1980- - >), Russian Science Citation Index (2005- >), SciELO Citation Index (2002- >)	'Yellow marmorated stink bug' OR '*Erthesina fullo*' OR 'Yellow spotted stink bug' OR 'YSSB' OR 'YMSB'	'Topic'	41

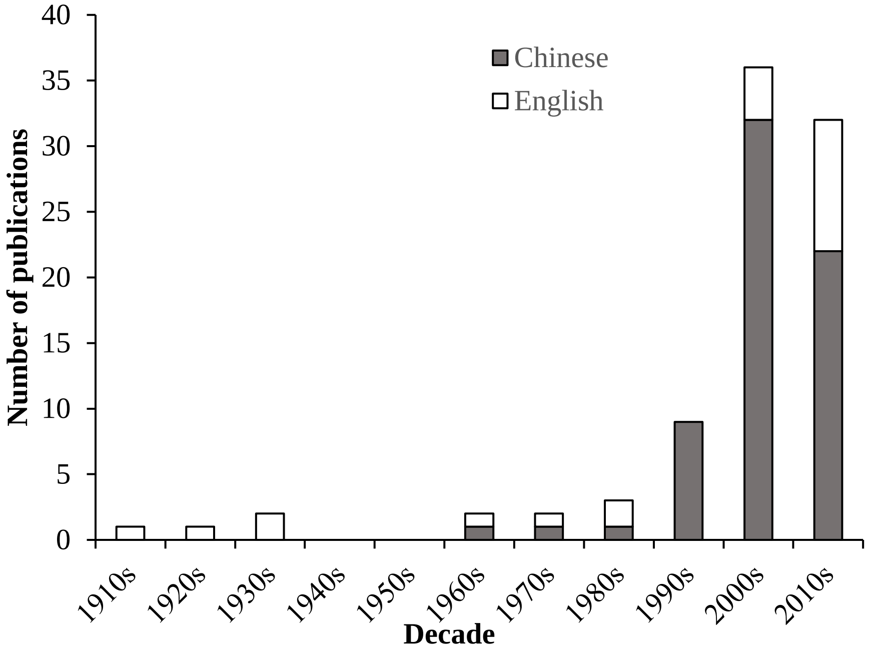

Figure S1. Number of publications on *Erthesina fullo* published by decade in Chinese and English.
